# Premenstrual syndrome among medical versus non-medical workers and its association with work-related quality of life

**DOI:** 10.1186/s42506-024-00161-z

**Published:** 2024-08-01

**Authors:** Nesma A. Mahmoud, Noha O. Frere, Nahla A. Zaitoun, Mai M. Zaitoun, Raghda A. Elshamy

**Affiliations:** 1 Department of Public Health and Community Medicine, Faculty of Medicine, Zagazig University, Zagazig, Egypt; 2 Department of Family Medicine, Faculty of Medicine, Zagazig University, Zagazig, Egypt; 3 Department of Obstetrics and Gynecology, Faculty of Medicine, Zagazig University, Zagazig, Egypt; 4 Department of Occupational Medicine, Faculty of Medicine, Zagazig University, Zagazig, Egypt

**Keywords:** Medical workers, Non-medical workers, Premenstrual syndrome, PMS, WRQL

## Abstract

**Background:**

Premenstrual syndrome (PMS) is a commonly underestimated disorder that negatively impacts a woman’s life. Medical workers, who live a more stressful life, may report an increased rate of PMS. Studies on the relationship between PMS and work-related quality of life for medical professionals are scarce, particularly in the Arab world. This study aimed to compare the frequency of PMS among medical versus non-medical workers at Zagazig University and to assess the association between PMS and their work-related quality of life.

**Methods:**

A comparative cross-sectional study was conducted. The sample population consisted of 48 medical and 48 non-medical female workers aged 18–45 years from Zagazig University. The two groups filled out a questionnaire with 3 parts: sociodemographic and occupational data, the Premenstrual Symptoms Screening Tool (PSST), and the Work-Related Quality of Life Scale (WRQL).

**Results:**

Severe PMS was reported in 45.8% of medical workers versus 20.8% of non-medical workers with a statistically significant difference between both groups (*p* = 0.009). Binary logistic regression showed that being a medical worker, clinical specialty, ≥ 8 years of work, ≥ 24 working hours per week, and having a non-set hourly schedule were predictors for severe PMS. PMS was found to be a statistically significant predictor of poor WRQL (*p* < 0.001). There was a highly significant negative correlation between the PMS score and the WRQL score (*r* =  − 0.302, *p* < 0.001).

**Conclusion:**

Among medical workers, PMS is more common and more severe, and WRQL is worse and negatively correlated with PMS. We suggest further studies with larger samples to prove this association and planning for public health programs to screen for and manage PMS among medical workers in our community.

## Introduction

Premenstrual syndrome (PMS) is a cycle of symptoms that occurs before menstruation and interferes with a woman’s work or lifestyle [1]. The American College of Obstetricians and Gynaecologists (ACOG) developed the diagnostic criteria for PMS, which stated that symptoms had to start no later than 5 days before menstruation for at least three cycles and terminate no later than 4 days after the period begins [2].

The three categories of PMS symptoms are behavior-related, psychological, and somatic. Appetite and libido changes are among the behavioral symptoms, along with fatigue, insomnia, and disorientation. Some of the psychological symptoms include irritability, anger, crying, worry, difficulties in concentration, and decreased self-esteem. Some of the physical symptoms include nausea, weight gain, edema, water retention, headache, breast tenderness, backache, abdominal pain, flatulence, and pain in the muscles and joints [3].

Premenstrual syndrome (PMS) and premenstrual dysphoric disorder (PMDD), a severe form of PMS, are two disorders associated with premenstrual symptoms. Both exhibit cyclical occurrences of various symptoms that are severe enough to impair day-to-day activities [4].

Research in Saudi Arabia revealed a significant prevalence of PMS at 78.5% [5]. Eighty percent of Jordanian women were affected, according to a different study [6], and the prevalence rates in Egypt ranged from 47.2 to 64.8% [7, 8].

Numerous studies have found that employees who reported some premenstrual symptoms had a significantly reduced quality of life, decreased job productivity and performance, impaired social and interpersonal connections, more frequent hospital visits, and higher absence rates [9, 10].

PMS in the workplace is under-researched, particularly in Arab countries. In the context of the Arabian workplace, a deeper understanding of PMS is crucial due to the high prevalence of PMS in Arab nations and the expenses linked to it. The objectives of this study were to compare the frequency of PMS among medical versus non-medical workers at Zagazig University and to assess the association between PMS and WRQL among those females.

## Methods

### Study design

A comparative cross-sectional study was conducted; it took 6 months from January to June 2023.

### The sample

Forty-eight medical and 48 non-medical female workers aged 18–45 years from Zagazig University were included following a simple random technique. The medical workers were from the faculty of medicine and university hospitals. Non-medical workers were a matched group from the same university. Our exclusion criteria were pregnancy, a known psychiatric illness or gynecological problem that may mimic or exacerbate symptoms of PMS, hysterectomy, or being on hormonal treatments, including oral contraceptive pills.

The open Epi version 6 statistics program was used to calculate the sample size under the following assumptions: The confidence interval was 95%, the precision degree was 80%, and the prevalence of PMS was 80.2% among medical professionals and 36% among non-medical professionals [6]. As a result, our sample was 48 workers in each group (medical and non-medical).

### Data collection

We collected data through self-reported questionnaires from both groups. The questionnaire consisted of 3 parts. The first covered sociodemographic and occupational data like duration of employment, number of working hours per week, and work pattern (set hourly schedule or non-set hourly schedule). Medical workers were asked about their specialty, and whether their work was academic teaching or clinical practice.

The second part was the Premenstrual Symptoms Screening Tool (PSST) [11], a 19-item questionnaire with two domains: the first domain comprises the 14 DSM-IV physical and psychological manifestations of PMS/PMDD, while the second domain is composed of five items assessing the functional impact of premenstrual symptoms. Each item is rated on a 4-point Likert scale from “not at all” to “severe”. Diagnosis of PMDD (severe form) requires: (1) the presence of at least five symptoms from the first domain, rated as moderate to severe; (2) at least one of the first four symptoms (anger/irritability; anxiety/tension; tearful/increased sensitivity to rejections; and depressed mood/hopelessness) must be rated as severe; and (3) severe functional impact, at least one item of the second domain rated as severe. Moderate PMS diagnosis is established by the following criteria: (1) at least five of the premenstrual symptoms of the first domain rated as moderate to severe; (2) the presence of at least one of the first four symptoms rated as moderate or severe; and (3) at least one item of the second domain rated as moderate or severe. Participants who do not fulfill any of these three criteria are classified as no/mild PMS [11].

The third part was the Work-Related Quality of Life Scale (WRQL) [12], the second edition was used (added as a supplementary file). It contains 32 questions and seven subscales, including job and career satisfaction (JCS) (questions 1, 3, 8, 11, 18, and 20), working conditions (WCS) (questions 13, 16, 22, and 31), general well-being (GWB) (questions 4, 9, 10, 15, 17, and 21), home-work interface (HWI) (questions 5, 6, and 14), stress at work (SAW) (questions 7, 19, 24, and 29), control at work (CAW) (questions 2, 12, 23, and 30), and employee engagement (EEN) (questions 26, 27, and 28). The overall quality of life associated with work was examined in Question 32. Responses were collected according to a Likert scale from 1 ‘strongly disagree’ to 5 ‘strongly agree’ Before determining the subscale scores, the negative items were reverse coded. The total score ranged from 32 to 160 with higher scores showing a better work-related quality of life. Workers were considered to have a good WRQL when they scored above the median of 80, and a bad WRQL when they scored below 80.

To anticipate potential data collection challenges and establish the required time for data collection, a pilot study with 10 participants (10% of the total study participants) was carried out. Following the pilot, no changes were deemed necessary, so the pilot sample was added to the main sample.

### Statistical analysis

The SPSS program version 25.0 [13] was used to analyze the gathered data. Using the Chi-square (*χ*2) test, qualitative data were compared and presented as frequencies and percentages. Binary logistic regression was used to examine potential PMS and WRQL predictors. The independent factors were working as a medical worker, specialty, duration of employment, working hours per week, shifts per month, non-set hourly work schedule, and severity of PMS. The link between PMS and the WRQL subscale scores was investigated using the Spearman correlation analysis. *P* values below 0.05 were deemed statistically significant, whereas values < 0.001 were deemed highly significant.

## Results

The average age of medical versus non-medical workers was 32.9 versus 26.9 years, with no statistically significant difference between both. Also, there was no statistically significant difference between the two groups regarding marital status and residence (Table 1). About 40% of medical workers had a master’s degree, while 72.9% of non-medical workers were only college graduates, with a highly statistically significant difference between the two groups (*p* < 0.001). More than 70% of medical workers were clinicians.

**Table 1:** Sociodemographic and occupational data of medical (*n* = 48) and non-medical (*n* = 48) workers, Zagazig University, Egypt, 2023

Characteristics	Medical workers *n* (%)	Non-medical workers *n* (%)	*χ2*	*P* value
Sociodemographic characteristics:				
Age				
< 32 years	21(43.7)	28(58.3)	2.04	0.15
≥ 32 years	27(56.3)	20(41.7)		
Educational level				
Graduate	16(33.3)	35(72.9)		
Master’s	19(39.6)	7(14.6)	15.2	< 0.001**
MD	13(27.1)	6(12.5)		
Marital status				
Married	14(29.1)	14(29.2)		
Single	26(54.2)	23(47.9)	0.65	0.71
Divorced	8(16.7)	11(22.9)		
Residence				
Rural	18(37.5)	20(41.7)	0.17	0.67
Urban	30(62.5)	28(58.3)		
Work-related characteristics				
Specialty				
Academic	13(27.1)	NA	-	-
Clinical	35 (72.9)			
Working hours/week^a^				
< 24	22(45.8)	40(83.3)	14.7	< 0.001**
≥ 24	26(54.2)	8(16.7)		
Number of work shifts/month				
< 8	12(25)	NA	-	-
≥ 8	36(75)			
Work pattern				
Set hourly schedule	15(31.3)	38(79.2)	22.3	< 0.001**
Non-set hourly schedule	33(68.7)	10(20.8)		

**p < 0.001 is highly statistically significant

^a^median was used as a cutoff

Regarding occupational data, there were highly statistically significant differences between medical and non-medical workers: 58.3% of medical workers versus 16.7% of non-medical workers worked for 8 years or more; 54.2% of medical workers versus 16.7% of non-medical workers worked 24 h per week or more; and 86.7% of medical workers had a non-set hourly work schedule versus only 20.8% of non-medical workers (Table 1).

**Fig. 1:**
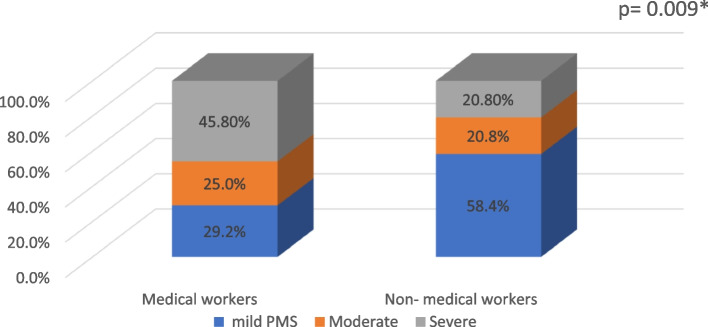
Frequency of premenstrual symptoms (PMS) among medical and non-medical workersat Zagazig University, Egypt, 2023

**Fig. 2:**
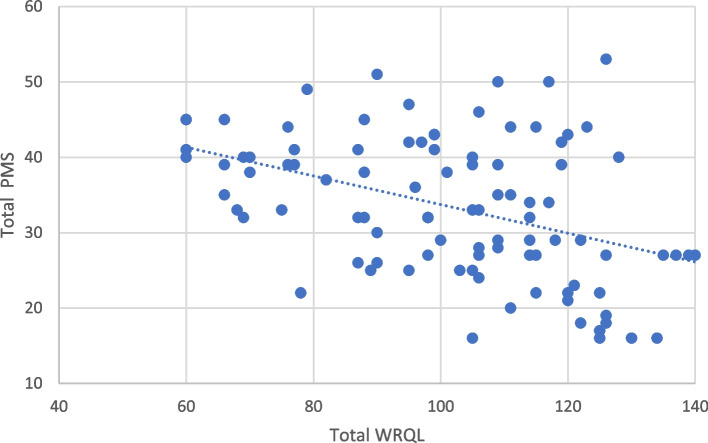
Correlation between PMS and WRQL subscales among medical workers (*r* =  − 0.302, *p < 0.001)

Results showed that all the items of the three PMS subscales were significantly different between both groups (Table 2). With regards to the degree of severity of PMS, 45.8%of medical workers versus 20.8% of non-medical workers had severe PMS, with a statistically significant difference between the two groups (Figs. 1 and 2).

**Table 2:** Premenstrual syndrome (PMS) symptoms among medical (*n* = 48) and non-medical (*n* = 48) workers

PMS	Medical workers *n* (%)	Non-medical workers *n* (%)	*χ2*	*P* value
Psychological-related symptoms^a^:				
Anger/irritability	30(62.5)	20(41.7)	4.11	0.04*
Anxiety/tension	28(58.4)	15(31.3)	7.11	0.007*
Tearful sensitivity to rejection	25(52.1)	10(20.8)	10.01	0.001*
Depressed mood	33(68.8)	20(41.7)	7.01	0.007*
Behavior-related symptoms^a^:				
Decreased interest in work activities	32(66.7)	18(37.5)	8.12	0.004*
Decreased interest in home activities	28(58.4)	18(37.5)	4.17	0.04*
Decreased interest in social activities	27(56.3)	15(31.3)	6.01	0.01*
Somatic-related symptoms				
Difficulty in concentration	34(70.8)	16(33.3)	13.51	< 0.001**
Fatigue	38(79.2)	22(45.8)	11.37	< 0.001**
Overeating	40(83.3)	26(54.2)	9.53	0.002*
Insomnia	24(50)	11(22.9)	7.61	0.005*
Hypersomnia	24(50)	10(20.8)	8.91	0.002*
Feeling overwhelmed	40(83.3)	22(45.2)	14.01	< 0.001**
Physical symptoms	36(75)	18(37.5)	13.71	< 0.001**
Severity of PMS				
Mild	14(29.2)	28(58.4)	8.29	0.003
Moderate	12(25)	10(20.8)	0.21	0.627
Severe	22(45.8)	10(20.8)	9.31	0.009*

*p < 0.05 is statistically significant

**p < 0.001 is highly statistically significant

^a^The sum does not add to 100 as multiple responses are allowed

There was a highly statistically significant difference between the two groups with regard to WRQL subscales. Regarding the total WRQL, 54.2%of medical workers versus 20.8% of non-medical workers had a bad WRQL, with a statistically significant difference between the two groups (Table 3).

**Table 3:** Work-Related Quality of Life (WRQL) among medical (*n* = 48) and non-medical (*n* = 48) workers

WRQL subscales	Medical workers *n* (%)	Non-medical workers *n* (%)	*χ2*	*P* value
Control at work (CAW)	17(35.4)	35(72.9)	13.5	< 0.001**
Employee engagement (EEN)	21(43.8)	37(77.1)	11.15	< 0.001**
General wellbeing (GWB)	15(31.3)	28(58.3)	7.11	0.007*
Homework interface (HWI)	20(41.7)	33(68.8)	7.13	0.007*
Job career satisfaction (JCS)	13(27.1)	28(58.3)	9.57	0.001*
Stress at work (SAW)	16(33.3)	30(62.5)	8.18	0.004*
Working conditions (WCS)	20(41.7)	36(75)	10.97	< 0.001**
Total WRQL				
Good (score > 80)	22(45.8)	38(79.2)	11.37	< 0.001**
Bad (score < 80)	26(54.2)	10(20.8)		

*p < 0.05 is statistically significant

**p < 0.001 is highly statistically significant

Univariate analysis assessing the association between socio-demographic, occupational characteristics, and PMS showed that working as a medical worker (*p* 0.01), specialty (*p* 0.02), longer duration of employment (*p* 0.007), more working hours per week (0.02), ≥ 8 shifts per month (*p* 0.01), and a non-set hourly schedule work pattern (0.01) were statistically significantly associated with the occurrence of severe PMS (nontabulated data).

To detect possible predictors for severe PMS, binary logistic regression showed that working as a medical worker (OR 23.4), clinical specialty (OR 5.33), working for 8 years or more (OR 2.97), working for 24 h per week or more (OR 3.85), non-set hourly work schedule (OR 3.4), and low WRQL(OR 2.76) were predictors for severe PMS (Table 4).

**Table 4:** Logistic regression analysis for detection of the possible predictors of PMS among all study participants

Predictors for PMS	*B*	S.E	Wald	AOR (95% CI)	*P* value
Working as a medical worker	3.1	0.81	16.0	23.4(4.92–59.63)	< 0.001**
Clinical specialty	1.67	0.45	13.50	5.33 (2.18–13.03)	< 0.001**
8 years of work or more	1.09	0.29	14.02	2.97 (1.68–5.27)	< 0.001**
24 working hours/week or more	1.35	0.29	20.36	3.85 (2.14–6.93)	< 0.001**
8 work shifts/month or more	1.64	0.49	3.99	2.69(0.94–2.75)	0.91
Non-set hourly schedule	1.22	0.43	7.98	3.40 (1.45–7.97)	0.005*
WRQL	1.97	0.57	14.4	2.76(1.99–6.98)	< 0.001**

*AOR* adjusted odds ratio

*p < 0.05 is statistically significant

**p < 0.001 is highly statistically significant

The association between socio-demographic and occupational characteristics and WRQL using univariate analysis showed that working as a medical worker, clinical specialty, more working hours per week, ≥ 8 shifts per month, and severe PMS were statistically significantly associated with WRQL.

To detect predictors for WRQL, binary logistic regression was done, controlling for other covariates. Working as a medical worker, clinical specialty and severe PMS were statistically significant predictors of WRQL [AOR were 29.4, 5.33, and 5.33 respectively] (Table 5).

**Table 5:** Logistic regression analysis for the detection of the possible predictors of bad WRQL among all study participants

Predictors for WRQL	*B*	S.E	Wald	AOR (95% CI)	*P* value
Working as a medical worker	3.3	0.62	29.2	29.4(8.06–100.11)	< 0.001**
Clinical specialty	1.60	0.41	13.50	5.33 (2.18–13.03)	< 0.001**
24 working hours/week or more	0.97	0.22	2.17	2.04(0.89–4.65)	0.41
8 work shifts/month or more	0.17	0.14	1.91	1.21(0.91–1.19)	0.19
Severe PMS	1.67	0.456	13.50	5.33(2.33–14.97)	< 0.001**

**p < 0.001 is highly statistically significant

Table 6 displays the WRQL and PMS correlation coefficients. The correlation between the PMS total score and the overall WRQL score was highly significant (*r* =  − 0.302, *p* ≤ 0.001). The overall WRQL score decreased when the PMS total score increased (Fig. 2). Furthermore, all subscale scores of the WRQL were negatively correlated with the PMS total score and subscales. Anger/irritability, anxiety/tension, and depressive mood were negatively correlated with General well-being, home-work interface, job career satisfaction, and stress at work. Tearful sensitivity to rejection was negatively correlated with employee engagement, general well-being, home-work interface, and stress at work. Decreased interest in work activities was negatively correlated with control at work, employee engagement, and job career satisfaction. Decreased interest in home and social activities was negatively correlated with general well-being. Difficulty in concentration was negatively correlated with all WRQL subscales except Job career satisfaction. Fatigue was negatively correlated with general well-being, home-work interface, and stress at work. Overeating was negatively correlated with the home-work interface. Insomnia, hypersomnia, and physical symptoms were negatively correlated with general well-being, homework interface, stress at work, and working conditions. Feeling overwhelmed was negatively correlated with control at work, job career satisfaction, and working conditions.

**Table 6:** Spearman correlation between the mean scores of PMS and WRQL subscales among medical workers

PMS	WRQL Subscales							Total WRQL
	CAW	EEN	GWB	HWI	JCS	SAW	WCS	
Anger/irritability	− 0.043	− 0.117	− 0.348*	− 0.199*	− 0.321*	− 0.301*	− 0.081	− 0.199*
Anxiety/tension	− 0.152	− 0.146	− 0.332*	− 0.276*	− 0.410*	− 0.391*	− 0.132	− 0.221*
Tearful sensitivity to rejection	− 0.054-	− 0.345*	− 0.029*	− 0. 391*	− 0.115	− 0.346*	− 0.122	− 0.207*
Depressed mood	− 0.072	− 0.108	0.411*	− 0.164*	− 0.412*	− 0.323*	− 0.062	− 0.317*
Decreased interest in work activities	0.430*	0.30–1*	− 0.021	− 0.143	− 0.186*	− 0.155	− 0.135	− 0.197*
Decreased interest in home activities	− 0.072	− 0.122	− 0.401*	− 0.152	− 0.042	− 0.123	− 0.149	− 0.177*
Decreased interest in social activities	− 0.114	− 0.123	− 0.328*	− 0.116	− 0.113	− 0.138	− 0.124	− 0.184*
Difficulty in concentration	− 0.414*	− 0.399*	− 0.321*	− 0.214*	− 0.099	− 0.309*	− 0.298*	− 0.311*
Fatigue	− 0.112	− 0.092	− 0.345*	− 0.277*	− 0.117	− 0.209*	− 0.103	− 0.243*
Overeating	− 0.113	− 0.112	− 0.146	− 0.305*	− 0.141	− 0.103	− 0.082	− 0.169*
Insomnia	− 0.139	− 0.102	− 0.298*	− 0.352*	− 0.122	− 0.287*	− 0.195*	− 0.302*
Hypersomnia	− 0.144	− 0.048	− 0.311*	− 0.399*	− 0.109	− 0.305*	− 0.223*	− 0.212*
Feeling overwhelmed	− 0.371*	− 0.100	− 0.122	− 0.133	− 0.167*	− 0.072	− 0.245*	− 0.276*
Physical symptoms	− 0.092	− 0.113	− 0.411*	− 0.277*	− 0.119	− 0.199*	− 0.278*	− 0.329*
Total PMS score	− 0.231*	− 0.211*	− 0.352*	− 0.225*	− 0.152*	− 0.296*	− 0.271*	− 0.302*

*CAW* control at work, *EEN* employee engagement, *GWB* general wellbeing, *HWI* homework interface, *JCS* job career satisfaction, *SAW* stress at work, *WCS* working conditions

*p < 0.05 is statistically significant

## Discussion

The relationship between PMS and work-related quality of life in medical staff as compared to non-medical staff has not been sufficiently studied, so we decided to assess the frequency and severity of PMS in medical workers and its effect on work-related quality of life.

There was a statistically significant difference in all work-related factors due to the stressful nature of medical work compared to non-medical work (more working hours and shifts, non-set hourly schedule). Additionally, medical workers are expected to be lifelong learners compared to non-medical university employees, which adds to the stressful nature of medical work.

We found that severe PMS affected 45.8% of medical workers using the Premenstrual Screening Tool. Compared to other studies, the study participants experienced a comparatively high frequency of PMS. In a study conducted on Thai nurses, the prevalence of PMS was reported as 25.1% [14]. According to the results of another Turkish study, 38.1% of nurses were diagnosed with PMS [15]. In a meta-analysis, the prevalence of PMS was estimated to be 47.8% across all study groups, ranging from 12% in France to 98% in Iran [16]. However, some studies in Arab countries found a similarly high prevalence, such as the Hammam et al. study [17], in Egypt which found that 66% of medical workers had PMS compared to 36% of non-medical workers. Another study [6] on Jordanian women found an even higher prevalence of PMS (80.2%). This shows that there are regional differences in the occurrence of PMS. PMS prevalence varies depending on cultural factors, sample size, sample composition, and the tool used to detect the disorder.

When compared to the overall PMS score, we found that the difference between the two groups was highly statistically significant (*P* < 0.001), with the total PMS score for medical workers being 40.01 and the score for non-medical workers being 32.09. This finding is supported by a study done in India by Selvarajan and Ajmal [18]. Although they used a different tool to assess PMS, they found a significant difference between medical and non-medical workers; PMS was more severe among medical workers. A study conducted in Taiwan by Cheng et al. [19] reported similar results.

This can be attributed to the higher levels of work stress that medical workers encounter, and according to Jahromi et al*.* [20], PMS may be exacerbated by work stress. To correlate work-related factors with PMS severity, binary logistic regression demonstrated that clinical specialty, working for 8 years or more, working for 24 h/week or more, and a non-set hourly schedule were all risk factors for moderate to severe PMS. This is in line with a study by Ahn et al. [21] in Korea that found stress at work, as well as working hours and environment, to be risk factors for PMS. Another study on working women in Korea found that factors like occupational characteristics (working hours and working type) enhance the likelihood of experiencing PMS [22].

The study’s findings showed that there was a statistically significant difference between medical and non-medical workers’ overall total WRQL. This finding is in line with a cross-sectional study that involved 15 hospitals and found that the majority of individuals had low WRQL and reported uninteresting work and dissatisfaction with the working environment [23]. As previously indicated, medical personnel had more shifts, longer working hours, and a non-set hourly schedule, which may explain their worse WRQL. According to Somsila et al. [24] working hours and the number of shifts have a significant impact on WRQL.

Binary logistic regression revealed that there were only three variables that were statistically significant predictors of WRQL: working as a medical worker, clinical specialty, and severe PMS. A similar regression was done by Sut et al. [15] and found that age and PMS score were predictors of WRQL.

Spearman correlation revealed a negative correlation between the PMS total score and overall WRQL; furthermore, all subscales of the WRQL scale were negatively correlated with PMS subscales. Consistent with this finding, Sut et al. also reported that there was a negative correlation between the total PMS score and the overall WRQL score [15].

Several other studies have found a relationship between PMS symptoms and poor work performance. For instance, Borenstein et al. [25] showed that women with PMS were less productive at work. Another study conducted in southern California confirmed that women's quality of life and productivity at work are greatly impacted by PMS symptoms [26]. A third study reported that affected work performance was reported by 70.3% of symptomatic healthcare personnel working at Dicle University Hospital in Turkey [27]. This can be explained by the fact that PMS raises tension, anxiety, and conflict-proneness, which in turn lowers quality of life and work performance [28].

### Limitations

The limitations of our study were the small sample size, the difficulty of generalization of the observed frequency as the sample was selected from a specific population, using a self-reported questionnaire without any clinical diagnosis, and the absence of assessment of work performance and absenteeism caused by PMS.

## Conclusion

Among medical workers, PMS is more common and more severe, and WRQL is worse and negatively correlated with PMS. We suggest further studies with larger samples to prove this association. We also recommend a national screening program for all women working in the medical field to assess their PMS symptoms and WRQL and offer different management strategies by a multidisciplinary team.

## Data Availability

The datasets used and analyzed during the current study are available from the corresponding author upon reasonable request.
